# Combined lateral peripatellar and posteromedial approaches for Schatzker type IV tibial plateau fractures involving posteromedial plane: a prospective study

**DOI:** 10.1186/s12891-020-03274-6

**Published:** 2020-04-13

**Authors:** Jun Zhang, Bo Yin, Jianmin Zhao, Yihan Li, Peng Yin, Tao Guo

**Affiliations:** 1 Department of Orthopaedics, The Affiliated Hospital of Innermongolia Medical University, Hohhot, 010010 China; 2 Department of Orthopaedics, Beijing Chao-Yang Hospital, China Capital Medical University, Beijing, 100020 China; 3 Department of Orthopaedics, Guizhou Provincial People’s Hospital, Guiyang, 550002 China

**Keywords:** Lateral peripatellar, Posteromedial, Tibial plateau, Fractures, Meniscus

## Abstract

**Background:**

The objective of this study to evaluate prospectively the effectiveness of Schatzker type IV tibial plateau fractures involving posteromedial plane managed by combined lateral peripatellar and posteromedial approaches.

**Methods:**

We analyzed 18 patients with Schatzker type IV tibial plateau fractures involving posteromedial plane. There were 12 males and 6 females with an average of 38.5 years (range, 25–60 years). The mechanism of injury included traffic accident in 15 patients and falling in 3 patients. The injured lower limbs were right in 11 patients and left in 7 patients. The mean time from injury to surgery was 6.78 days (range, 5–9 days). There were 8 patients with meniscus injuries in our study.

**Results:**

The mean operation time was 3.41 h (range, 3–4 h). The mean blood loss was 352.78 ml (range, 300–410 ml). All the injured meniscuses were repaired. All patients were followed up, and the average time of follow up was 16.61 months (range, 14–22 months). Bone union was achieved at a mean of 12 weeks (range, 10–14 weeks). The mean degree of knee extension was 1.11° (range, 0–5°), and the mean degree of knee flexion was 120.56° (range, 110–130°). The mean points of KSS were 83 (range, 74–89 points). According to the criteria of KSS, 14 patients had clinical outcomes rated as excellent and 4 patients were rated as good.

**Conclusion:**

Our results suggested that Combined lateral peripatellar and posteromedial approaches in the treatment of Schatzker type IV tibial plateau fractures involving posteromedial plane acquired satisfying outcomes. It was good for repairing the injured meniscus through our approaches.

## Background

Tibial plateau fractures are most commonly described according to the Schatzker classification system based on location, morphology and treatment. Schatzker type IV fractures are described as the fractures involving the medial plateau [1]. They account for 10–30% of all tibial plateau fractures [2, 3]. The type IV fractures commonly result from high-energy trauma, and usually accompany with associated injuries of meniscus and ligaments [3, 4], Therefore, the treatment is still difficult in clinical practice, especially the fractures involving posteromedial plane.

At present, we can choose either medial or posteromedial approach to explore the site of Schatzker type IV tibial plateau fractures, but either of them has its limited visualization of the articular surface of the medial plateau [5]. Especially in the fractures involving posteromedial plane, the two approaches are often unable to achieve the sufficient reduction because of the limitation of visualization, and the meniscus cannot be elevated through the approaches if the meniscus is trapped in the fracture line. The type of the fracture is not uncommon. Yang et al. reported the fractures involving posteromedial plane accounted for 42.7% of Schatzker type IV fractures [1].

Therefore, in order to solve the limitation of visualization, we added a lateral peripatellar approach as viewing window to aid in congruent joint reduction. To our best knowledge, no study about the combination of lateral peripatellar and posteromedial approaches in the treatment of Schatzker type IV tibial plateau fractures involving posteromedial plane has been reported. In the following report, we shared with our successful experience in the management of Schatzker type IV tibial plateau fractures involving posteromedial plane through the combination of lateral peripatellar and posteromedial approaches.

## Methods

Between January 2015 and January 2016, 42 patients with Schatzker type IV tibial plateau fractures were treated in our institution. All the operations were performed by the same orthopaedic surgeon. The inclusion criteria were: (1) the fractures involved posteromedial plane; (2) Patients of age of 18 years or more; (3) The injured knee joint was normal before injury. The exclusion criteria were: (1) Open or pathological fractures; (2) a history of knee surgery; (3) Fractures complicated by serious nerve or vascular injury. According to the inclusion and exclusion criteria, 18 patients were included in our study. Our study was approved by the Chinese PLA General Hospital committee for clinical research and informed consent was obtained from the 18 patients.

There were 12 males and 6 females with an average of 38.5 years (range, 25–60 years). The mechanism of injury included traffic accident in 15 patients and falling in 3 patients. The injured lower limbs were right in 11 patients and left in 7 patients. The mean time from injury to surgery was 6.78 days (range, 5–9 days). There were 8 patients with lateral meniscus injuries in our study. More details listed in Table 1.

**Table 1:** Demographic characteristics of 18 patients with Schatzker type IV tibial plateau fractures involving posteromedial plane

Case number	Sex	Age (years)	Mechanismof injury	Injured lower limbs	The time from injury to surgery (days)	Meniscus injuries
1	Male	35	TA	Right	7	Yes
2	Male	30	TA	Right	6	No
3	Female	28	TA	Left	8	Yes
4	Male	40	TA	Right	7	Yes
5	Male	45	TA	Right	7	No
6	Male	33	TA	Left	9	No
7	Female	56	F	Left	5	No
8	Female	38	TA	Right	7	Yes
9	Male	25	TA	Right	8	Yes
10	Male	60	F	Right	5	No
11	Female	31	TA	Right	7	No
12	Male	45	TA	Left	7	No
13	Male	39	TA	Left	6	Yes
14	Female	33	TA	Left	5	No
15	Male	43	TA	Right	7	No
16	Male	37	TA	Left	6	Yes
17	Male	30	TA	Right	9	Yes
18	Female	45	F	Right	6	No

*TA* traffic accident, *F* falling

### Surgical technique

**Fig. 1:**
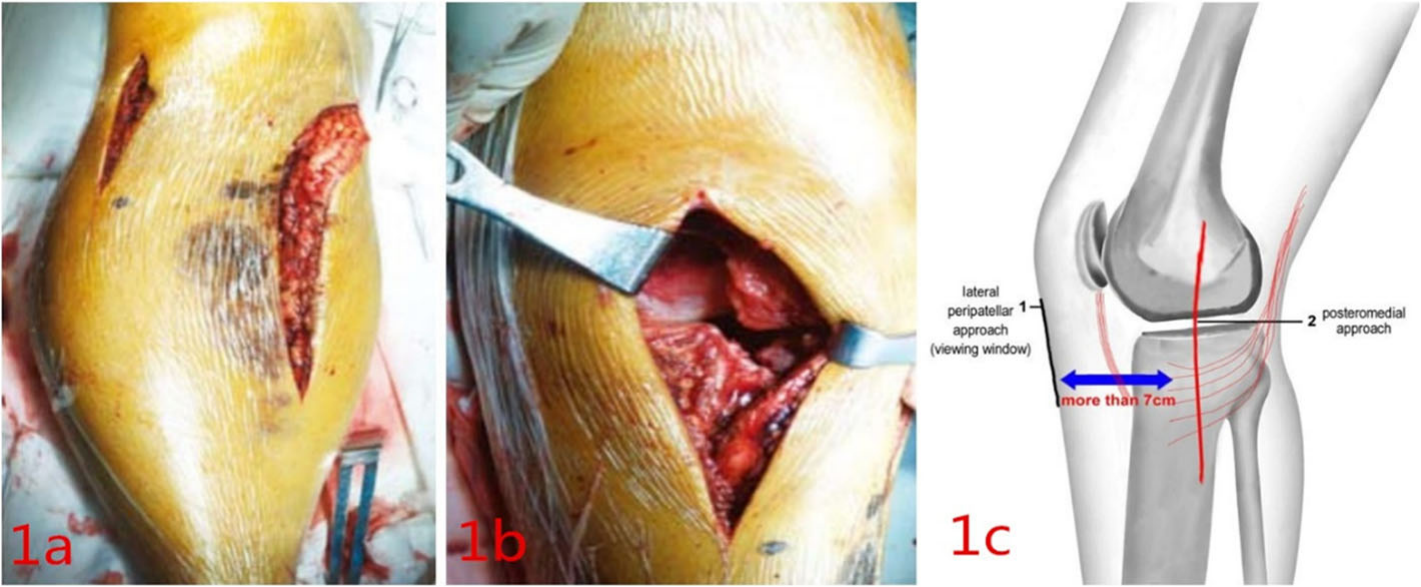
Incision design **a** Asmall incision was made on the basis of posterior medial approach and assisted by lateral parapatellar approach **b** After cutting the retaining band, the patella and patellar ligament were pulled medially to clearly expose the intra-articular. **c** Schematic drawing of combined lateral peripatellar and posteromedial approaches. The distance between the two skin incisions should be more than 7 cm

The patient was positioned supine on a radiolucent table under intratracheal general anesthesia. A pneumatic tourniquet was applied to the proximal thigh. Posteromedial approach was performed firstly [6–8]. A longitudinal incision was made along the posteromedial aspect of the tibia. The saphenous nerve and the great saphenous vein should be protected during exposure. Then the dissection was performed between the semitendinosus muscle and the medial head of the gastrocnemius muscle in order to explore the pes anserinus tendon. The fracture line usually existed at the level of pes anserinus tendon at the metaphysis. The pes anserinus tendon was cut off along the incision of skin, and it was marked in order to repair it after the surgery. During the course of cutting off the pes anserinus, we should pay more attention in the protection of MCL (medial collateral ligament, MCL). When the site of fracture was exposed, the lateral peripatellar approach as viewing window was performed to aid in congruent joint reduction. The small incision (About 4-5 cm) was made along the lateral peripatellar (The distance between the two skin incisions should be more than 7 cm as the Fig. 1 showed), and then peripatellar retinaculum was incised. The patella and ligamentum patellae were pulled medially so that we could visualize the injured articular surface directly. If the meniscus is trapped in the fracture line, we can detect its injury severity and make its repair through the lateral peripatellar approach. When the anatomical reduction of the articular fracture was achieved, a medial buttress plate and a posterior plate were used at the apex of the fracture. Incisions were then closed over suction drains.

### Postoperative care

A knee plaster was performed, if the patient had an associated injury of ligament. Dressings were changed in 48 h and drainage tubes were pulled out 48 or 72 h after the surgery. Soft tissues were closely monitored in 48 h in order to avoid the compartment syndrome. The passive range-of-motion exercise was started at the first day after the operation. The full activity of knee was finished in 4–6 weeks after the surgery and progressive weight-bearing was started at 10–12 weeks after the operation.

### Evaluation of outcome

Operation time, blood loss, bone union time, and the degrees of knee extension and flexion were indices of the outcome. The final clinical outcomes of knee were evaluated by Knee Society Score (KSS) [9, 10]. The outcomes were categorized as excellent (80–100 points), good (70–79 points), fair (60–69 points), or poor (< 60 points).

## Results

**Fig. 2:**
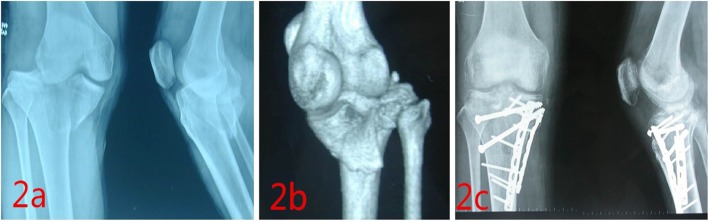
The figure showed a 35-year-old man (case 1) who had the Schatzker type IV right tibial plateau fracture involving posteromedial plane. **a** The radiograph of the patient before the operation. **b** The three-dimension reconstruction image of the patient before the operation, and it showed the Schatzker type IV tibial plateau fracture involved posteromedial plane. **c** The fracture was fixed by dual plates, with one plate fixing the medial fragment and one fixing the posterior fragment. The avulsion fracture of ACL was fixed by lag screws

The mean operation time was 3.41 h (range, 3–4 h). The mean blood loss was 352.78 ml (range, 300–410 ml). All the injured meniscuses were repaired. All patients were followed up, and the average time of follow up was 16.61 months (range, 14–22 months). All patients underwent anteroposterior and lateral knee X-ray examination at one month, three months and six months after operation. Bone union was achieved at a mean of 12 weeks (range, 10–14 weeks). The mean degree of knee extension was 1.11° (range, 0–5°), and the mean degree of knee flexion was 120.56° (range, 110–130°). The mean points of KSS were 83 (range, 74–89 points). According to the criteria of KSS, 14 patients had clinical outcomes rated as excellent and 4 patients were rated as good (Fig. 2). More details listed in Table 2. There were no serious complications in our study. One patient had transient palsy of saphenous nerve (Case 4), and he was recovered completely at 10 weeks after surgery. One patient had superficial wound infection (Case 8), and she was cured by oral antibiotics.

**Table 2:** Clinical data of 18 patients with Schatzker type IV tibial plateau fractures involving posteromedial plane

Case number	Operation time (hours)	Blood loss (ml)	Follow-up (months)	Bone union time (weeks)	Knee extension/ flexion (°)	Knee Society Score (points)	Outcomes
1	3.2	350	16	12	0–0-120	86	Excellent
2	3.5	370	14	10	0–0-125	84	Excellent
3	3	330	14	12	5–0-130	88	Excellent
4	3.6	400	18	14	0–0-115	77	Good
5	4	410	16	12	0–0-125	80	Excellent
6	3.5	340	22	12	0–0-115	83	Excellent
7	3	300	15	14	0–0-110	74	Good
8	3.4	360	17	12	0–0-120	80	Excellent
9	3.2	360	14	12	0–0-125	86	Excellent
10	3.4	330	16	14	0–0-110	79	Good
11	3.7	380	18	12	5–0-120	85	Excellent
12	3.2	340	15	10	0–0-125	89	Excellent
13	3.9	380	19	12	5–0-120	87	Excellent
14	3.3	340	16	10	0–0-130	84	Excellent
15	3.6	350	18	12	0–0-125	83	Excellent
16	3.2	330	17	12	0–0-115	79	Good
17	3.5	360	18	12	0–0-120	86	Excellent
18	3.1	320	16	12	5–0-120	84	Excellent

## Discussion

This is the first prospective study of combined lateral peripatellar and posteromedial approaches in the treatment of Schatzker type IV tibial plateau fractures involving posteromedial plane. The present study showed that the approach acquired satisfying clinical outcomes. All patients had clinical outcomes rated as excellent or good. The motion ranges of the knee joint were nearly normal levels. All patients achieved bone union on time.

The main goals in the treatment of tibia plateau fractures include anatomic reduction of articular surface, maintenance of normal knee alignment, provision of knee stability and restoration of a painless range of motion and function [4, 11–13]. Schatzker type IV tibial plateau fractures involving posteromedial plane remain difficult, because they usually accompany with meniscus and ligaments injuries. Two approaches have been applied to expose the type of fracture. However, either of them has its limitations. The medial approach with a medial parapatellar hasn’t given an adequate exposure of medial plateau, and it is very difficult to fix the posteromedial fragment if we don’t make an extensive dissection of the soft tissue. The posteromedial approach allows an wider exposure of posteromedial fragment for the fixation of the buttress plate [14–16]. However, it is unable to elevate and repair the lateral meniscus that trapped in the fracture line due to the limited visualization. Stannard et al. reported that there was a 49% meniscus injury during high-energy tibial plateau fractures, and the incidence of lateral meniscus injury was more than medial meniscus injury [17]. Barrow et al. reported the incidence of meniscus injury was 25% in Schatzker type IV tibial plateau fractures of their study [18]. Therefore, in order to solve the limitation of the two approaches, we designed the combined lateral peripatellar and posteromedial approaches. It could not only aid in the anatomic reduction of articular surface, but also repair the injured meniscus.

**Fig. 3:**
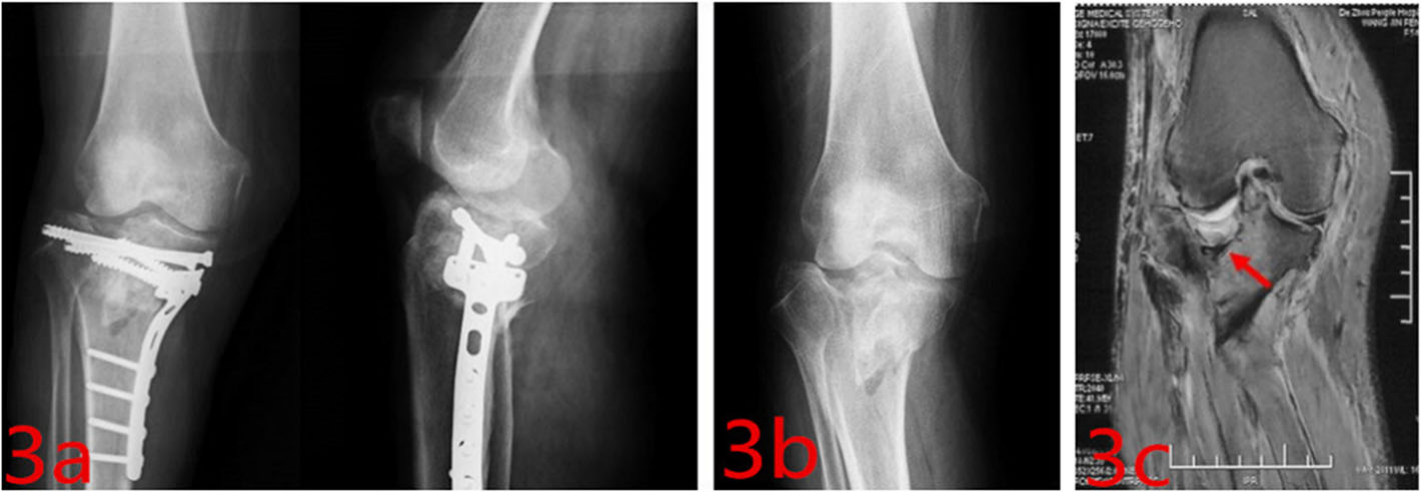
This is a failure case about restoring the width of plateau through the medial approach in other hospital. **a** Post-operative images showing that the width of plateau was not restored. **b** Pre-operative images. **c** The MRI showed the meniscus was trapped in the fracture line

The incidence of injured meniscus was 44.44% (8/18) in our study. We repaired all the injured meniscuses when we achieved the adequate fixation of the fracture by dual plates. We believed that it was very important to repair these injured meniscuses, because they could allow patients to perform the early functional exercises and be beneficial to restore the normal ranges of knee motion. There were no serious complications in our study. Only 5.56% (1/18) patients had transient palsy of saphenous nerve and 5.56% (1/18) patients had superficial wound infection, and finally they were all cured. We thought we could overcome these complications as long as we pay more attention to them. The main advantage of our approach was that it could overcome the limited visualization to aid in the anatomic reduction of articular surface and repair associated injured menisci at the same time. Especially in the case of failure of restoring the width of plateau through the medial or posteromedial approach (Fig. 3), we should consider if the injured meniscus is trapped in the fracture line or not and add a lateral peripatellar approach as viewing window to aid in congruent joint reduction. However, the view of medial tibial plateau through the lateral window is very limited for the middle and posterior third of the medial meniscus.

In our experience, some important aspects should be paid. (1) We should evaluate carefully the fracture and the soft tissue envelope based on specific conditions of the patient. Handing the soft tissue properly in this region is critical to successful treatment. (2) Computed tomography (CT) is necessary before the operation. Magnetic resonance imaging (MRI) is recommended, because it can provide information with regard to associated meniscus and ligaments injuries that may affect the treatment plan. (3) We should try our best to repair the injured meniscuses. There are two types of fracture combined with cruciate ligament injury. The first is the avulsion of cruciate ligament. Ligament’s bony avulsions should be repaired at the time of the internal fixation, acutely, thus promoting better prognosis due to direct bone to bone healing. The second is the rupture of the cruciate ligament. We should repair it through arthroscopy after the healing of fracture. As for MCL (medial collateral ligament, MCL) or LCL (lateral collateral ligament, LCL) injury, we also shouldn’t add an additional to repair the injured MCL or LCL, and we need add a knee plaster to protect the injured ligaments, because MCL or LCL damage is often incomplete. (4) In our approaches, the distance between the two skin incisions should be more than 7 cm in order to avoid the necrosis of the skin. (5) The incision of the lateral peripatellar approach should be small, because it just is a viewing window to aid in the treatment of the fracture.

The main strength of our study is that all the operations were performed by the same orthopaedic surgeon, and it can avoid the differences caused by different surgeons’ preference and experience. The type of the study is prospective. However, there are some limitations. The number of patients is relatively small, and control group isn’t included in our study. Therefore, the potential biased on our outcomes may be exist, and more patients need to be included in the future study to verify the effectiveness of this procedure and overcome the limitations of our current outcomes.

## Conclusions

In conclusion, our study suggested that combined lateral peripatellar and posteromedial approaches in the treatment of Schatzker type IV tibial plateau fractures involving posteromedial plane acquired satisfying outcomes. It was good for repairing the injured meniscus through our approaches.

## Supplementary information

**Additional file 1.**

## Data Availability

The data used to support the findings of this study are included within the article. All data and materials were in full compliance with the journal’s policy.
